# United Nations sustainability development goals approached from the side of the biological production of fuels

**DOI:** 10.1111/1751-7915.13912

**Published:** 2021-08-24

**Authors:** Ana García‐Franco, Patricia Godoy, Jesús de la Torre, Estrella Duque, Juan L. Ramos

**Affiliations:** ^1^ Estación Experimental del Zaidín CSIC Granada E‐18008 Spain; ^2^ Programa de Bioquímica y Biología Molecular University of Granada Granada Spain

## Introduction

Huge economic and technological leaps have been made since the start of industrial revolution in the 18th century and through developments in the last 70 years. Improvements in the production and quality of goods, increases in food production and advances in medicine have contributed to enhance human life expectancy across the world. The increase in human population, with the trend of people moving from rural zones to cities, the vision of large companies for instant global business and, the development and expansion of terrestrial, maritime and air transportation have led to a highly connected world. The current COVID pandemic has exacerbated the global universe and the ‘internet of things’ has arrived and plans to stay. During the first two decades of the 21st century, it has been estimated that terrestrial transport represents nearly 20% of all carbon emissions, and CO_2_ emissions linked to aircraft were calculated to be 2% of all human emissions (www.eee.europa.eu; Becken and Mackey, 2017). The continuous increase in CO_2_ in our atmosphere with current levels of 420 ppm – about 20% higher than 50 years ago, and other greenhouse gases has given rise to a silent pandemic. Some aircraft builders (e.g. Boeing) are planning to fly using 100% sustainable aviation fuels by 2030 and several companies have reported successful testing of biofuel aircraft (www.biofuels‐news.com/Feb 2021).

Global climate change could devastate our environment if a point of no return is reached, this would result in a shortage of food that leads to massive malnutrition, famine and, eventually end our civilization. Many voices, governmental and non‐governmental, are asking for measures to ameliorate first the current situation and then to restore conditions that are compatible with sustainable life.

The Kyoto Protocol and the more recently the Paris Climate Agreement call for the use of clean, green and renewable fuels to replace fossil fuels in all transportation areas (United Nations, 2016). The overall goal is to reduce petroleum energy dependence by more than 80% by 2050, a goal which requires multiple approaches – eolic‐, thermo‐solar, photovoltaic, wave power, bioenergy and others forms of renewable energy. No single means of making energy is sufficient to cover the world’s energy demands (Chen *et al*., 2017); however, when developed and used concurrently, these approaches may be sufficient to meet current and future demands. These alternative sources of energy can be sourced from sunlight (i.e. photovoltaic and thermosolar), wind, ocean tides, plant biomass and microbes (Hussain *et al*., 2017).

Significant efforts are being made to reduce the Carbon footprint related to transportation and a number of measures are being taken to introduce electric and hybrid cars, hydrogen propelled trucks, trains and other heavy vehicles. However, due to the nature of the different means for transportation (terrestrial, maritime, air), different types of fuels have to be considered. Liquid biofuels for transportation already represent an alternative to replace not only part of the fossil hydrocarbons but also as a means to save carbon emissions and reduce toxic net emissions linked to gasoline and diesel combustion motors. Below, we analyse how developments in bioethanol comply with UN SDGs, and provide suggestions for more clean energy generation.

### Implications of biofuels for Sustainable Development Goals (SDGs)

In the late 1980’s Gro Harlem, Norwegian prime Minister and chair of the Commission on Environment and Development, defined Sustainable development as ‘development that meets the needs of the present without compromising the ability of future generations to meet their own needs’ (https://en.unesco.org/themes/what‐is‐esd). However, it took until 2015 for the adoption of 17 multilateral and international SDGs in the 2030 Agenda for Sustainable Development, which were approved at the UN SDG summit held in New York in the fall of 2015 (https://sustainabledevelopment.un.org; United Nations, 2015). Although measures to reach SDGs are not compulsory for governments, it is true that some Governments are making efforts to design policies to reach them, unfortunately the rate of adoption of said measures is not sufficient such that the desired objectives will be reached by 2030 (Bexel and Jonsson, 2016; Barbier and Burgess, 2017). The current concept of Sustainable Development encompasses three interconnected loops: economic development, social inclusion and environmental sustainability (United Nations, 2015). The SDGs aim to ameliorate global warming, the most obvious damage of which are those from natural disasters caused by unusual atmospheric phenomena for example intensive rains and floods and extreme dry spells. The less obvious of which are unexpected biological events such as plagues, diseases and virus expansion like the current COVID pandemic (Brüssow, 2020, 2021).

Biofuels are produced from biological materials, most often oils, cereal grains, sugarcane or biomass derived from plants or wastes, and they represent an alternative to fossil fuels that offers a number of social, economic, environmental and technical benefits (Koçar and Civaş, 2013; Voegele, 2013; Ramos *et al*., 2016; Valdivia *et al*., 2016; Ramos and Duque, 2019). The main drivers behind biofuels are: (i) energy supply security and reduction in fossil oil use (SDG 7: Clean energy); (ii) support of rural areas through technology development and new jobs based on technology (SDG 2, 8 and 9), (iii) mitigation of global GHG emission and reduction of particulate materials that are toxic for humans, animals and plants (SDG 7). Therefore, biofuels can contribute towards the responsible use of energies and the replacement of a fraction of fossil fuels by one of the available green renewable sources (https:www.eca.europa.eu). Achievement of SDGs requires holistic action, however, they must be deconstructed and analysed at the sector level to define how industrial activities can be modified to favour the achievement of SDGs.

Biofuels are considered renewable fuels because they are derived from plant materials that are made through photosynthesis and CO_2_ fixation; processes which in principle reduce net emissions of GHG. However, just because a biofuel derives from CO_2_, fixation is not sufficient to declare it a viable alternative. The rules established by Hill et al. (2006) for ‘a biofuel to be a viable fossil‐fuel alternative should be considered; namely, a viable biofuel must provide a net energy gain, have environmental benefits, be economically competitive and be produced in large amounts without reducing food supplies’ (Hill *et al*., 2006). Therefore, for a specific biofuel, the total carbon sequestered by plants must compensate for all the emissions linked to its production and manufacturing (Hill *et al*., 2006). Furthermore, the full life cycle has to be considered so that in term of emissions its production should count direct and indirect emissions derived from changes in land use, the amount of carbon sequestered and the amount of greenhouse gases emitted (Crutzen *et al*., 2008; Mosier *et al*., 2009, 2014). In general, achieving carbon neutrality for biofuels requires high plant yields and low emissions. The so‐called first generation ethanol produced from cereals was the subject of controversy because the UN considered that as a consequence of derivation of cereals to ethanol the cereal prices for human consumption increased. In addition, fears arose because the use of agricultural land for biofuel production could endanger food production, these issues have been and are part of the ‘food versus fuel’ debate.

Nonetheless, the field of bioethanol production in particular and biofuels in general, is an exceptionally dynamic and exciting arena and this industry can contribute positively to a number of the SDGs. Together they can help to reduce poverty (SDG 1); reduce fossil oil dependency for energy and because combustion of biofuels is cleaner than fossil fuels lead to a reduction in net toxic emission is achieved (SDG 7); through the use of agricultural residues and municipal solid wastes support a circular economy (SDG 2 and 3); facilitate land restoration and promote the use of land and marginal lands to grow energy crops, which leads to the creation of high‐qualify and stable rural jobs (SDG 8 and 13); they can also promote industrial development and specialized job creation (SDG 9). In the case of ethanol, the world production of bioethanol is estimated (gallons per year) to be about 15 billion in the United States, 6 billion in Brazil and about 3 billion in Europe; which is equivalent to the replacement of about 750 million barrels of petroleum every year. Below we analyse bioethanol, the largest fermentative process for a chemical commodity in the context of UN SDGs.

SDG1: ‘reduce poverty’. The petroleum market is highly volatile due to limited reserves and the distribution of these reserves in countries with unstable political situations. Reducing dependency of oil and gas supply from a few countries by self‐produced renewable energies will help to recover world equilibria and create new resources for countries adopting renewable energy approaches, which in turn will help to reduce poverty (Gielen *et al*., 2019). At present, advances in the area of biofuels take place in more developed countries, particularly the United States, however, industrial plants located in other countries which are capable of generating energy are feasible – governments need to support their stable operation and educate the population to advance these solutions. Guaranteed energy supply promotes advances in the primary sector and will support industrial development, which in turn contributes to reduce poverty.

SDG 2: ‘Zero hunger’. Goal 2 aims to end hunger and all forms of malnutrition by 2030. It also commits to universal access to safe, nutritious and sufficient food all times of the year. This requires sustainable food production systems and resilient agricultural practices, equal access to land, technology and markets and international cooperation on investments in infrastructure and technology to boost agricultural productivity (htpps./www.theexplorer.no/goals/zero‐hunger/. www.jordantimes.com).

Bioethanol, produced from corn grain (or other cereals) in the United States, Europe and Asia, and sugar cane (Brazil), became controversial because the UN concluded that food prices increased due to biofuel production. Although currently the corn used to produce biofuels are non‐edible varieties, the land used for grain production for biofuel competes for land for grain for human use, and hence we have to take it as a negative factor because the so‐called first‐generation (1G) bioethanol is mainly made from food crops grown on arable land (Mohr and Raman, 2013). 1G ethanol producers have implemented a series of technological advances to reduce environmental impact, that is through harvest of CO_2_ for medical or industrial uses, corn oil recovery for human consumption and the use of the resulting solid residues – known as dry distillers’ grain (DDG) – that is used as animal feed, because it is rich not only in fibre but also in protein and vitamins from the yeasts used to ferment sugars in the ethanol production process.

SDG 7: Energy is crucial for achieving almost all of the Sustainable Development Goals, from its role in the eradication of poverty through advancements in health, education, water supply and industrialization, to combat climate change (htpps./www.theexplorer.no/goals/affordable‐and‐clean‐energy/).

Bioethanol is the most relevant biologically produced commodity. Almost all of the ethanol used in the world for pharma, solvent industries and fuels is produced through biological fermentation. The so‐called 1G bioethanol fuel is produced worldwide, although the main producers are Brazil and the United States. Ethanol is produced through the fermentation of sugars derived mainly from corn grain and sugarcane, as well as from sugar beet, wheat grain (or other cereal grains), molasses and various other plants, including fruit and fruit waste. In the case of grain, the first step of biofuel production is the hydrolysis of starch using amylases. This process produces simple sugars – mainly glucose – which are then fermented to ethanol using microorganisms such as yeasts or bacteria (e.g. *Zymomonas*). In the United States, ethanol production rates are in the range of 14–15 billion gallons per year at corn dry mills and this technology is quite mature and industrial ethanol plants are usually profitable in the United States. In Brazil, around 5 billion gallons of ethanol are produced annually and the leftover waste (i.e. bagasse) is often burnt in the mills to generate extra energy.

SDG 9: ‘Industry, innovation, and infrastructure, which aims to build resilient infrastructure, promote inclusive and sustainable industrialization and foster innovation’. Infrastructure provides the basic physical systems and structures essential to the operation of a society or enterprise. Industrialization drives economic growth, creates job opportunities and thereby reduces income poverty. Innovation advances the technological capabilities of industrial sectors and prompts the development of new skills (htpps./www.theexplorer.no/goals/industry‐innovation‐and‐infrastructure/; www.unstats.un.org).

Innovation and Development in the field of bioethanol started with an attempt to address the food versus fuel controversy that forced the bioethanol industry to search for new feedstock. Biomass was considered the most immediate source for bioethanol, and likely other chemicals (Duque *et al*., 2018; Pandey and Prakash, 2018). This gave rise to the concept of cellulosic ethanol or 2G bioethanol that can be made from corn stover, sugarcane straw, wheat straw and other agricultural wastes, as well as the organic fraction of municipal solid waste (MSW) (Schwartz, 2010).

Because 2G ethanol technology is based on byproducts of other crops (i.e. food crops on arable land), it opens significant opportunities to the whole manufacturing chain, including farmers, the ethanol industry, new biotechnology companies, project developers and investors. However, this sector is not mature and still requires significant industrial improvements before it can become a consolidated process. Obviously, an added‐value of second‐generation biofuels is the avoidance of competition for food producing land and the reduced need to use additional water or fertilizers.

To place the biofuel contribution to SDG9 in context, we should consider that the production of 2G bioethanol involves three major actions: (i) the physicochemical pre‐treatment of the biomass that destroys plant structure and makes biopolymers available (ii) the enzymatic breakdown of cellulose and hemicellulose into constituent sugars to provide abundant glucose (~ 70%) and xylose (~23%); and (iii) fermentation of these C6 and C5 sugars using specialized yeasts (Taherzadeh and Karimi, 2007; Alvarez *et al*., 2016). Several physico‐chemical pre‐treatments efficient in deconstruction of plant materials are in place and no major developments are pending except in the area of biomass handling. The main source of enzymatic cocktails for cellulose and hemicellulose are enzymes secreted by fungi. Relevant developments in the area of 2G technology are still needed (Sharma *et al*., 2020). The degradation of cellulose and hemicellulose requires a number of enzymes that work cooperatively to breakdown cellulose and hemicellulose through the action of endocellulases followed by a number of exo‐cellulases that release oligosaccharides that through the action of glucosidases and xyloxidases yield monomeric sugars. Industrial enzymatic cocktails commercialized by Novozymes or Dupont enable the release of > 80% of the sugars in celluloses and hemicelluloses (Alvarez *et al*., 2016).

The area of enzymatic hydrolysis has achieved very good results with herbaceous material, but even so, the enzyme costs represent up to 25% of the operation costs and with woody biomass, the enzyme loads required are higher which in turn creates and even larger deficit (Valdivia *et al*., 2016). It is clear that investment in enzyme technology is required. Advances will likely come from the use of thermophilic enzymes and from new discovery programs that approach the issue from the metagenomic angle. Specific cocktails deficient in one or more functions are available to identify more robust and better performing enzymes for each of the critical steps. Other improvements will come the generation of improved proteins through site‐directed or random mutagenesis.

The enzymatic reactions required take place in an acidic environment and at high temperatures. These two requirements are relevant as they reduce potential contamination by microorganisms. A source of relevant enzymes are fungi from extreme environments and the development of ‘wholesale’ consensus design of thermodynamically stable proteins (Sternke *et al*., 2019) and metagenomic analysis (Duque *et al*., 2018). Although using symbiont cocktails made of fungi and bacterial enzymes, such as those that occur in nature, may be feasible to reduce costs of the enzymes. Once some of these steps are resolved the biofuel sector can promote the development of auxiliary industries to produce the enzymes.

Fermentation of sugars released from corn stover, sugarcane straw and other agricultural residues are rich in C6 and C5 sugars, but because most *Saccharomyces* do not ferment C5 sugars and a number of recombinant yeasts have been constructed that are capable of simultaneously fermenting these sugars (Heer and Sauer, 2008; Caballero and Ramos, 2017). These recombinant organisms can transform more than 96% of glucose, and more than 90% of xylose into ethanol, and when endowed with arabinose fermentation capability > 90% of the theoretical maximum – an achievement that demonstrates how far this technology has progressed.

Current advances in yeast fermentation strains are directed at incorporating some enzymes on the yeast cell surface, which can enhance the amount of sugars available for fermentation or inactivate inhibitors. These improvements in the yeast sector can be straight forward and new yeast strains relatively easily obtained. As such, the production of yeast biomass for 2G technology can be considered a relevant accessory industry.

Butanol can replace gasoline in internal combustion engines. Butanol has been produced at an industrial level through the anaerobic process of ABE fermentation carried out by different strains of *Clostridium*. The standard fermentation yields a mixture of acetone, butanol and ethanol (1:6:1) (Green, 2011), but butanol production can be increased to represent up to 80% of the product (Jang *et al*., 2012). Techno‐economic studies support that biobutanol is more profitable than bioethanol and that the capital expenses (CAPEX) required to upgrade ethanol plants to butanol can be returned in 4–5 years depending on the plant production capacity. A number of Clostridia strains are able to degrade lignocellulose material (i.e. from agricultural waste and forestry residues.), suggesting that a second‐generation biobutanol industry is feasible (Wen *et al*., 2021).

The conversion of municipal solid waste (MSW) into bioethanol is extremely challenging. Kalago *et al*. (2007) estimated that conversion of the organic fraction of municipal solid waste to fuels may save up to 16% of all of the fuel used in the transport sector in the United States. Production of bioethanol from a 160 million tons of MSW per year would provide 7.5 billion gallons of ethanol with a saving of 250 million petrol barrels. In addition, the conversion of the organic fraction into ethanol will save emission of greenhouse gases to the atmosphere.

SDG 13 climate action. Take urgent action to combat climate change and its impacts. Climate change presents the single biggest threat to development, and its widespread, unprecedented impacts, disproportionally burden and most vulnerable. Urgent action to combat climate change and minimize its disruptions is integral to the successful implementation of SDGs (htpps./www.theexplorer.no/goals/climate‐action/) (www.unoosa.org.).

In order to reduce external oil dependence and save petrol reserves, the United States mandated to mix gasoline and ethanol many years ago, the mixture commonly known as E10 gasoline contains a 10% ethanol blend with gasoline. This blend can be used in conventional vehicle motors without modifications and it has been established that it reduces particulate matter emissions, and benzene, toluene and xylene emissions – the most toxic compounds in car/truck exhaust, but in turn, the levels of NO_x_ emissions increase (Niven, 2005).

SDG 15: Life on land. ‘Protect, restore, and promote sustainable use of terrestrial ecosystems, sustainably manage forests, combat desertification, and halt and reverse land degradation and halt biodiversity loss. Preserving diverse forms of life on land requires targeted efforts to protect, restore and promote the conservation and sustainable use of terrestrial and other ecosystems. Goal 15 focuses specifically on managing forests sustainably, restoring degraded lands and successfully combating desertification, reducing degraded natural habitats and ending biodiversity loss’. (htpps./www.theexplorer.no/goals/life‐on‐land/).

The use of marginal soil for energy crop production has been analysed by Somerville *et al*. (2010); the process requires rapid adaptation of plants to water‐limited lands and the selection of varieties that thrive under semi‐arid/arid conditions (Carroll and Somerville, 2009). Successful implantation requires not only accurate estimations of the energy density per hectare and the potential biofuel to be produced but should also take into account another less obvious aspect; that plants will transfer 20% of the CO_2_ fixed to soil which in part will be stabilized acting as a carbon sink. This exudate carbon will be a source of nutrients to develop biodiversity and will help restoration of soils (Timmis and Ramos, 2021; Ramos and Timmis, 2021). The growth of roots represents a relevant form of Carbon sequestration and exudates non‐consumed carbon may react with mineral components of soil facilitating its slow metabolism.

The utilization of marginal soils for energy crop production will enhance available soil, and in the mid‐term, may make soils useful for edible crops. Energy crop plants are grown specifically for biofuel production, but the crops vary with geography (Koçar and Civaş, 2013; Hood *et al*., 2013). For example, in the United States, the most cultivated energy crop plants are corn, soybeans, willows and switchgrass (Cseke *et al*., 2009); while in northern Europe preferential crops are rapeseed, wheat, sugar beet and willows. Sugarcane is the main energy crop grown in Brazil with sugar being fermented and bagasse burnt in combined cycle plants. While the promising *Miscanthus* is grown in Southeast Asia. We envisage new research programs to create more resistant and better adapted energy crop plants that can be grown in marginal soils.

## Conclusions and perspectives

Despite the potential held by biofuels, at present only about 1% of the total energy used globally comes from biofuels, and in the transport sector only about 3% of the world’s fuel for road transport is of biofuel origin (Sharma *et al*., 2020). As such, there are extensive opportunities to increase the use of renewable fuels. In order to reduce dependency on petroleum, several international agencies and governments are aiming to use biofuels to supply 25% of their transportation energy by 2050, although current trends to use hydrogen to propel trucks may change this number. We should consider that the value of biofuels goes beyond their use as transportation fuels, and attention should be given to the economic and environmental benefits of the co‐products, plus the saving of raw chemicals for production of thousands of every day products (Yang and Wyman, 2008; Yuzbashev *et al*., 2010; McKenna and Nielsen, 2011; Yang *et al*., 2012; Liu *et al*., 2013; Philp *et al*., 2013; Akhtar *et al*., 2014; Ramos and Duque, 2019). The replacement of fossil fuels by 1G and 2G ethanol reduces greenhouse gas emissions, and decreases the need for crude oil. In turn, it aligns with UN SDGs that encourage the use of renewable energy sources. Furthermore, bioethanol is linked to agricultural crops and in this sense, it promotes the creation of rural jobs. Presently, the long‐term success of 2G ethanol and biochemicals requires further research, financial incentives to help create mature ethanol production processes and supportive regulations, which are instrumental for driving the commercial production and adoption of advanced biofuels.

The Lignin challenge: In the 2G process a semisolid cake remains after the process ends, this includes the untouched lignin, the rest of the fibres, non‐degraded sugars and other residues. The most immediate use of this cake is to burn it to generate electricity. However, the greatest value is in the lignin, a heteropolymer that can be processed to produce new chemicals, and potentially start a new chemistry Rasgaukas *et al*., 2014). A number of enzymes including laccases, ligninases and others are being explored in an effort to release lignin monomers as raw material or modified lignin multimeric rings to create new products (Mate and Alcalde, 2017). The decade to come should reveal lignin potential; if this happens the value of the 2G bioethanol industry will reside in exploitation of the residues to add further value. At which point, 2G technology will then contribute to SGD 3 in the recycling of products.

## Funding Information

Work in Granada was supported by MINECO‐FEDER RT2018‐094370‐B‐I00 research project.

## Conflict of interest

None declared.
